# Study on the Aging Characteristics of a ±500 kV Composite Dead-End Insulator in Longtime Service

**DOI:** 10.3390/polym16131944

**Published:** 2024-07-08

**Authors:** Zhijin Zhang, Bingbing Wang, Xuze Li, Shude Jing, Yuan Gao, Dong Zeng, Xingliang Jiang

**Affiliations:** Xuefeng Mountain Energy Equipment Safety National Observation and Research Station, Chongqing University, Chongqing 400044, China; wangbb@stu.cqu.edu.cn (B.W.); Lyxuze@stu.cqu.edu.cn (X.L.); jingshude@stu.cqu.edu.cn (S.J.); gaoyuan@stu.cqu.edu.cn (Y.G.); 20231101007@stu.cqu.edu.cn (D.Z.);

**Keywords:** flashover characteristics, hydrophobicity, electrical characteristics, FTIR, TGA

## Abstract

Composite insulators have been widely used in power grids due to their excellent electrical-external-insulation performance. Long-term operation at high voltage levels accelerates the aging of composite insulators; however, there is a scarcity of research on aged composite insulators operating at 500 kV for over ten years. In this paper, the mechanical, electrical, and microscopic properties were tested on different sheds along a 500 kV composite insulator that had been running for 18 years. Additionally, the results were compared with a new insulator and the standards for live insulator operation. The results showed that the aging of the high-voltage end of composite insulators was the most serious. The results of the physical properties test indicated that the insulator’s hardness was compliant but its tensile strength and break elongation did not meet standards. Under wet conditions, the pollution flashover voltage decreases by about 50% compared to the new insulator. Combined with the microscopic test results, the shed skeleton structure could be damaged and the filler might be lost during the aging process of polydimethylsiloxane (PDMS). The hardness of the insulator would increase by the precipitation of inorganic silicon; however, inorganic silicon might destroy the hydrophobicity and other properties of insulator sheds. These results can provide theoretical references for insulator life prediction and operation protection.

## 1. Introduction

Composite insulators have been widely utilized in China’s power system due to their excellent hydrophobicity and pollution-flashover-resistance characteristics [1,2,3]. HTV silicone rubber (SIR) is the main material used in the composite insulator shed. The chemical composition of SIR is based on the Si-O bond, which works as the main chain, and the hydrophobic methyl or vinyl groupworks as the branch chain [4]. According to previous research, the aging phenomenon of SIR is caused by environmental stress factors, such as ultraviolet light, humidity, and temperature. Further, the aging of SIR can lead to serious disadvantages, such as chalking (where certain particles of fill form a rough or powdery surface), damage, and hydrophobicity decrement [5,6,7,8]. Since insulators operate in the outdoor environment, their performance is greatly affected by environmental stress factors. These potential issues will affect not only the stable operation of composite insulators but also the reliability of the power system. Therefore, it is of great significance to study the aging state and aging patterns of SIR and to conclude the deterioration law of composite insulators, which will help ensure the safety and reliability of transmission networks.

The insulator sheds are an important and functional component of composite insulators. To enhance the reliability of composite insulators, researchers worldwide have investigated the aging phenomenon of insulator sheds. Some studies are focusing on the aging characteristics under different years and natural environments. Mavrikakis et al. [9] analyzed the hydrophobicity of aged 380 kV composite insulator samples with a service life of more than ten years and found that the insulator surface had become hydrophilic. This resulted in increased electric field intensity in certain areas, which exceeded the corona onset threshold and led to corona discharge on the insulator surface. Additionally, the corona further degraded the surface and accelerated the aging process. Zhou et al. [10] compared the difference in reflectivity of the Si-O-Si main chain and Si-CH_3_ side-chain in an insulator shed. The study concluded that long-term pollution and ultraviolet radiation were the primary causes of aging. Zhang et al. [11] found that the performance improvement of composite insulators by de-chalking treatment was inconsistent, and the chemical composition of the surface after removal of the chalking layer could be restored to a state similar to the SIR. Mu L et al. [12] observed that as the service time of composite insulators increased, the hardness of composite insulators increased. The increase in micropores and filler precipitation can lead to decreased tear strength, potentially damaging the shed and reducing insulation strength.

Researchers have studied the aging characteristics under the artificial aging tests conducted in the laboratory. Yao et al. [13] selected various SIR materials to undergo corona discharge aging tests and analyzed the impact of corona discharge on the aging properties. Liang et al. [14,15] explored how factors such as contamination degree, aging duration, and environmental humidity affected the hydrophobicity and flashover voltage of SIR subjected to corona aging; meanwhile, the influence of surface discharge properties on flashover voltage was also analyzed in the study. Zhang et al. [16] investigated the mechanical properties before and after the experiment, revealing that the surface discharge became stronger with the increase in the applied voltage, leading to a reduction in hydrophobicity, degradation of surface properties, and a continued degradation in the mechanical properties of SIR exposed to acidic conditions. Jiang et al. [17] studied the fog-flashover characteristics of polluted insulators under different conductivity salt-fog environments using the artificial pollution test method. Wang et al. [18] studied the flashover characteristics of post insulators in a haze environment by simulating the haze environment. Scholars have extensively researched the corona aging characteristics of SIR and the flashover characteristics of composite insulators; however, the various aging methods and environments can introduce deviations in the test results.

The aging condition of composite insulators operating under high voltage for extended periods remains unclear, especially for those operating at 500 kV and above for over ten years [19,20,21]; moreover, the impact of mechanical and microscopic changes in the pulverized insulator shed on the power system is uncertain. Therefore, it is important to study the pulverization phenomenon of composite insulators. This article visually inspected the appearance status of 500 kV composite insulators that operated for 18 years. Furthermore, a natural pollution accumulation test and pollution flashover test were carried out, as well as detailed hydrophobicity, mechanical properties, and a pollution degree test. Upon comparing with the insulator operation standards and the new SIR sample, the reasons for the overall decline in the external insulation performance of composite insulators were revealed. This study could provide valuable insights for predicting the lifespan of insulators and optimizing operational protection measures.

## 2. Experiments

### 2.1. Sample

The composite insulator sample in this paper was from a specially customized non-standard strain tower. The hanging network model was 500/210-5950, with 16 insulators per tower arranged in 66 groups of varying sizes. It had a total tonnage of 21 tons, a creepage distance of 18,000 mm, and a creepage specific distance of 3.0 cm/kV. In the test insulator, the sheds at the high-voltage end, the middle part, and the grounding end were marked as A, B, and C, respectively. Additionally, the new SIR sample, labeled D, was used as a control sample for comparative testing.

### 2.2. Test Methods

**Figure 1:**
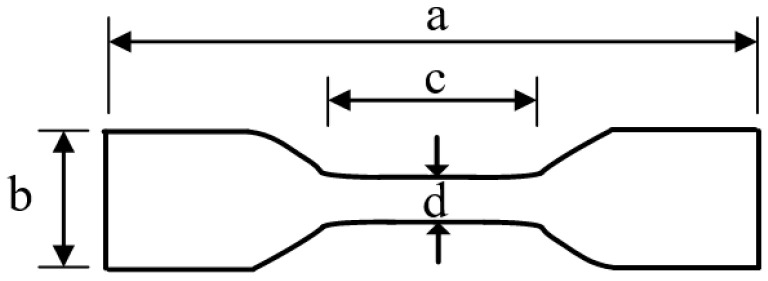
Dumbbell-shaped specimen: a—35 mm; b—6 mm; c—10 mm; d—2 mm.

- Visual inspection
- Visual inspection primarily assessed the aging of insulators by evaluating the color variation and the extent of damage in the insulator sheds;
- Roughness test
- The TR-200 roughness meter was utilized to measure the roughness of the samples, adhering to GB/T 1031-2009 [22]. Distinct areas on the insulator shed samples of A, B, C, and D, were selectively tested. Each selected area underwent 6 measurements, and the results were averaged to obtain a reliable roughness value;
- Hardness test
- The hardness of the samples was tested using the LX-A Shore hardness tester, following the ISO 7619-1 standard [23]. This standardized method involves selecting 5 distinct points on the surface of the sample and measuring their hardness using the Shore hardness tester. The average value of these five measurements provides the final hardness result;
- Hydrophobicity (HC) test
- The hydrophobicity of the composite insulator sample was evaluated before and after de-pulverization using the HC spray grading method. The experiment was conducted strictly following the requirements of DL/T 810-2012 [24];
- Tensile strength test
- The tensile strength and elongation at the break of the composite SIR samples were tested according to the ISO 37-2005 standard [25]. The sample was cut into a dumbbell-shaped piece using a JZ-6010 punching stage, with dimensions shown in Figure 1. The tensile testing machine utilized in this experiment featured an HLD scale spiral bracket, a digital display unit HP-500N, a frame stroke of 180 mm, a scale stroke of 150 mm, and a table load capacity of 500 N;
- During the measurement, the cross-section of the sample was evenly stressed, ensuring a measurement accuracy of ±2%. If the sample did not break at the narrowest point during the test, the result was discarded and replaced by a new sample test result. The final result was obtained by calculating the arithmetic average of the four measurements taken from each group;

- Natural pollution accumulation test
- The non-soluble deposit density (*NSDD*) and equivalent salt deposit density (*ESDD*) were tested according to the relevant regulations and procedures of GB/T 16434-1996 [26];
- Electrical characteristics test
- Flashover voltage tests were carried out under dry, wet, and salt-spray pollution conditions, respectively, and each sample underwent three flashover voltage tests. The interval between each test was approximately 1 to 2 min. The lowest flashover voltage value among the three tests was recorded as the flashover voltage for that sample. The average flashover voltages obtained from three test samples under the same contamination condition were taken as the representative flashover voltage for that state. The breakdown characteristics of insulators refer to IEC 60243-1 [27]. The sample was cut to 3.5 × 3.5 × 2.5 mm^3^, and the electrode pieces were clamped on both sides of the breakdown point. Transformer oil was selected as the insulation medium;
- Thermogravimetric analysis (TGA) test
- The test sample was cut into equal slices, and the powder was removed. The samples before and after treatment and the new sample slices were used for the TGA test. The thermogravimetric analyzer used in this study was the TGA/DSC1/1600LF, with air as the carrier gas, a heating rate of 10 °C/min, and a heating range of 30 to 650 °C. The tests were conducted under standard atmospheric voltage and room temperature;
- Fourier-transform infrared spectroscopy (FTIR)
- The Nicolet iS50 Fourier-transform infrared spectrometer was utilized in this paper to measure the infrared spectrum of the sample. Through FTIR testing, the chemical groups on the sample’s surface were identified, and further analysis was conducted to examine the trends in the content of these groups during the reduction aging process.

## 3. Results

### 3.1. Physical Properties

#### 3.1.1. Visual Inspection

After observation, the overall fading of the test sample string is severe, and the aging and whitening phenomenon is obvious. The whitening area is covered with a large number of easily shaken powders and a large number of cracks. The whitening of the high-voltage end and the middle part of the sample is more obvious and the whitening area was larger than that of the grounding-end area.

**Figure 2:**
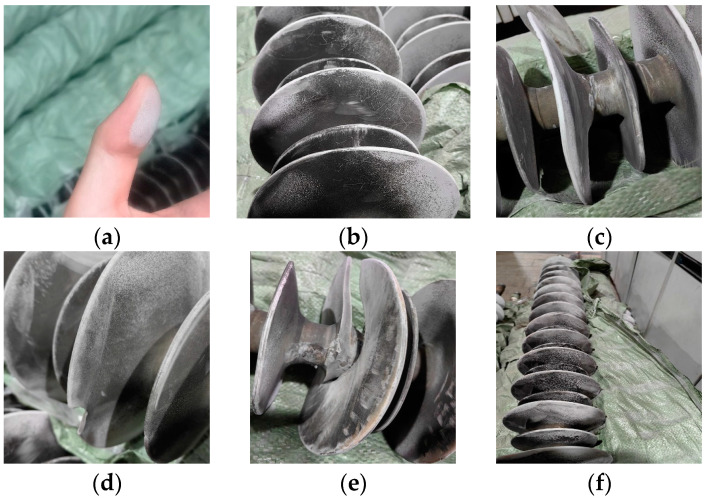
Visual inspection of insulator shed: (**a**) surface chalking of insulators; (**b**) small cracks on the surface; (**c**) changes in insulator structure; (**d**) small cracks on the edge of the insulator shed; (**e**) insulator shed fracture; (**f**) half of the insulators are severely chalking, while the other half is heavily dirty.

It can be seen that the sample insulator string is covered with a large amount of pollution, and the proportion of polluted sheds is more than 98%. The pulverization phenomenon on the surface is serious. The pulverization and whitening areas of each insulator shed were mostly distributed on the edge of the insulator shed. The closer to the core rod, the less obvious the chalking phenomenon is. At the same time, the orientation of the pulverization area is more consistent. Moreover, the proportion of cracked sheds in the sample has exceeded 80%, and the sheds have become seriously crisp and brittle, and the sheaths have been cracked and separated, as shown in Figure 2.

#### 3.1.2. Roughness

After the aging of composite insulation materials, micro-cracks often appear on the surface, causing the filling material to gradually escape from the cracks; consequently, the material becomes uneven, and its roughness significantly increases. Therefore, roughness is commonly used as an indicator to evaluate the degree of aging. The roughness test results of the samples are shown in Table 1.

The data in Table 1 show the following:

The three tested aging samples showed large differences in roughness across various regions, and the highest value of *Ra* was 27.131. In addition, the maximum *Ra* values of samples A, B, and C were more than 20, while the roughness value of the area covered with thicker natural contamination was relatively small, in the range of 2 to 5. Compared with the new sample D, the roughness value of the aged sample is relatively larger.

Upon visual inspection, it can be observed that half of the natural contamination in the sample is thicker, while the other half is obviously powdered. The position with the largest roughness has many cracks and serious pulverization, and, almost no smooth surface can be found in that position.

After longtime operation, certain areas of the surface of the aged insulator shed have serious chalking and cracks, which results in a significant growth in the roughness values of these areas. Meanwhile, the natural contamination also increases the roughness of the insulator.

#### 3.1.3. Hardness

The increase in the hardness of the insulator shed is often accompanied by a decrease in mechanical properties, creating a major safety hazard. The hardness measurement results of the samples are shown in Table 2.

The above results indicate the following:

The value of hardness of the aged sample is about 70–90 HA, while the value of hardness of the new sample D is about 66 HA, which means that the value of hardness of the aged sample is greater than that of the new sample. Furthermore, DL/T 376-2010 stipulates that the mechanical properties of SIR insulation materials should meet the Shore hardness requirement of not less than 50 Shore A. Based on the test results, it can be seen that the value of hardness of the aged sample meets the standard requirements. According to the hardness test, the longer the aging time, the greater the value of hardness of the insulator shed, so the value of hardness is not necessarily better when higher. At present, the relationship between the service life and the value of hardness of the shed is still unclear.

Sample A has the highest value of hardness among all the samples. The FTIR result below shows that the PDMS main chain of the high-voltage-end SIR is seriously broken, the chemical bonds Si-O and Si-CH_3_ in the polydimethylsiloxane were broken, and the silicone small molecules were lost. This degradation process contributes to the increased surface hardness observed in the SIR.

#### 3.1.4. Hydrophobicity

**Figure 3:**
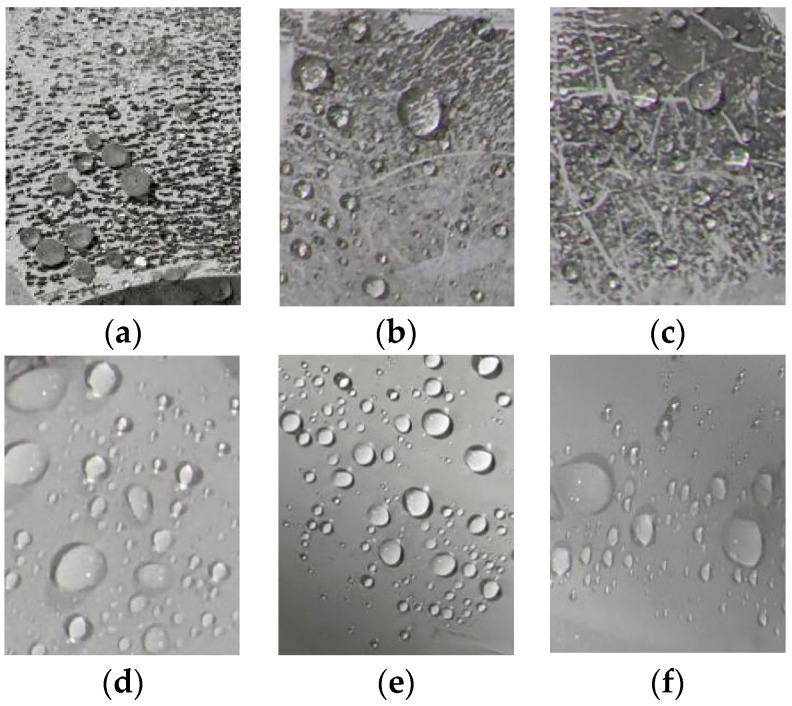
Sample HC level measurement: (**a**) sample A powdered surface; (**b**) sample B powdered surface; (**c**) sample C powdered surface; (**d**) sample A de-powdered surface; (**e**) sample B de-powdered surface; (**f**) sample C de-powdered surface.

The measurement results of the HC spray grading method are shown in Figure 3.

From the test results, the following can be seen:

The chalking surface of the samples has only separated water droplets. Most of the water droplets have small retreat angles, which means the hydrophobicity of the aged chalking samples belongs to the HC1 level. After removing the powder from the sample, the retreat angle of the water droplets decreases, and most of the water droplets are no longer circular. The hydrophobicity of the de-powered surface of samples A and C belongs to the HC3 level, and the hydrophobicity of the de-pulverized surface of sample B belongs to the HC2 level.

It is required in the standard DL/T 810-2012 [24] that the clean surface of three samples should generally be HC1 to HC2 grade, and there should be no more than one sample of HC3 grade. Therefore, the hydrophobicity of the chalking samples meets the standard requirements, while the insulator sheds after removing the powder do not meet the hydrophobicity requirements specified in this standard.

According to the above results, it can be seen that the chalking layer will not let the hydrophobicity degradation of the composite insulator; however, rain, ice, snow, and many other weather factors could result in the chalking layer peeling in live operation when the hydrophobicity of the material does not meet the standard requirements. Especially, the hydrophobicity of the high-voltage ends and grounding ends of operating insulators is relatively poor.

Combined with the FTIR results below, the degree of hydrophilicity between the surface of SIR and water molecules was analyzed. The results showed that the electric field of sample A is strong. The chemical bonds on the surface of SIR were broken, and a hydrolysis reaction occurred, which resulted in an increase in hydroxyl -OH. Since -OH is a hydrophilic group, the hydrophobicity becomes weak. Due to the serious aging of the high-voltage end, methyl-CH_3_ is also reduced. Because -CH_3_ is non-polar, the SIR will polarize, resulting in weaker hydrophobicity.

#### 3.1.5. Tensile Strength and Elongation at Break

The tensile strength and elongation at the break of the insulators are summarized in Table 3.

From the test results, the following can be concluded:

The difference in tensile strength and elongation at break of the four positions on the same sample is small. The slight difference may be due to the different degrees of hardening and brittleness of the samples near the mandrel position and the edge of the shed.

The tensile length and elongation at break of sample A are significantly smaller than those of samples B and C. The overall trend of tensile strength and elongation at break is consistent. The aggravation of surface aging of the insulator shed is often accompanied by the phenomenon that the material of the shed becomes harder and brittle, and the toughness decreases; therefore, from the analysis of mechanical characteristics, it is considered that the aging of the high-voltage end of a dead-end composite insulator is more serious.

DL/T 810-2012 [24] requires that the mechanical breaking strength of the shed material for insulators should not be less than 4.0 MPa, and the elongation at break should not be less than 150%. The tensile strength and elongation at break of the samples were lower than the critical value. Consequently, it is considered that the tensile mechanical properties of the tested sample can no longer meet the operating requirements.

### 3.2. Electrical Characteristics

#### 3.2.1. Natural Pollution Accumulation Test

The *NSDD* and *ESDD* test results of different parts of the tested sample are shown in Table 4. *T/B* is the pollution nonuniformity in Table 4, which is the ratio of the pollution distributed on insulator top sheds and bottom sheds.

From Table 4, it can be seen that the *ESDD* of the upper surface is slightly higher than that of the bottom surface, which may be because the sample is a dead-end insulator rather than a suspended insulator. The result distribution of the *ESDD* and *NSDD* of the sample string indicates that more pollution is accumulated at the high-voltage and grounding ends, and less in the middle of an insulator. The ratio of *NSDD* to *ESDD* is defined as the *NSDD/ESDD* value. The *NSDD/ESDD* value of most transmission lines is between 20 and 0.1, while the *NSDD/ESDD* value of the samples in this paper is above 20, and the maximum value is 50.12. According to previous studies, the insoluble pollution on insulators has an obvious influence on the flashover voltage; therefore, the influence of *NSDD* should also be fully considered when classifying the pollution level.

The insulator sheds experience corona and arc discharge, which make certain particles of fill form a rough or powdery surface. The powder and the natural contamination formed contamination particles, resulting in severe pollution contamination. Meanwhile, the presence of the towers affects the flow field near the insulator, leading to a reduction in the loss rate of contamination particles. Combined with the FTIR result, it is speculated that the non-soluble pollution contains a large amount of PDMS oxidative degradation substances in addition to natural fouling, and the ATH filler precipitated from the interior with the aging of the SIR and the Al_2_O_3_ is produced by its degradation.

#### 3.2.2. Breakdown Characteristics

This article adopts the even-raising method for the experiment, which uniformly boosts the voltage until the inside of the sample is broken down. The experiment results are shown in Table 5. *E_b_* is the breakdown voltage per unit length, and *σ* is the standard deviation in Table 5. The relative deviation of the results was less than 10%, indicating that the results in this paper have a relatively small dispersion; therefore, the analysis based on experimental results has a certain rationality.

From the test results, the following can be seen:

The breakdown strength of samples decreased. The breakdown strength of sample A is 11.248 kV/mm, which is only 53% of sample D. Sample C maintains the best insulation performance, which is 77% of the new sample D.

According to GB/T 1695-2005, when the test medium is transformer oil with a spacing of 2.5 mm, the breakdown voltage should not be less than 35 kV, that is, the breakdown strength of the insulator should be greater than 14 kV/mm. The breakdown strength results show that the sample A can no longer meet the standard requirements. Although the breakdown strength of the middle and grounding end of the sample string has decreased, it still meets the standard requirements.

Based on the TGA analysis in the following text, the weight loss in the first and second stages of sample A is lower than that of samples B and C. The SIR in long-term live operation undergoes ATH decomposition and PDMS cracking, and the aging at the high-voltage end is more severe.

#### 3.2.3. Flashover Characteristics

In this paper, the even-raising method is used for the test, which uniformly boosts the voltage until the surface flashover of the sample. The test results are shown in Table 6. *E_f_* is the flashover voltage per unit length, and *σ* is the standard deviation in Table 6. The relative deviation of the results is less than 10%, indicating that the results in this paper have a small dispersion; therefore, the analysis based on the test results has a certain scientificity.

From the test results, the following can be concluded:

As for the same sample insulator, the flashover voltage of dry flashover, wet flashover, and pollution flashover were gradually reduced; moreover, the flashover voltage results of samples A and C are similar and both higher than that of sample B. The flashover voltage of the aged sample is about 97–99% of sample D, indicating that the flashover characteristics of the aged insulator do not decrease much in the dry flashover state.

Under wet-flashover and pollution-flashover conditions, the flashover voltage of sample A is less than that of samples B and C, and is 49.7% and 47.8% of the flashover voltage of sample D, respectively. The results indicated that sample A was more likely to have flashover accidents during operation. Combined with the above physical and microscopic characteristics results, it can be concluded that the high-voltage end of the tension pulverization insulator is seriously aged.

The flashover voltage of sample C is 12.7% higher than that of the middle under wet flashover and pollution flashover. In the natural pollution accumulation test, the *ESDD* of sample C is higher than that of sample B. It can be speculated that the surface contamination and pulverization layer are lost in the wet environment. Combined with the results of the TGA and FTIR analyses, the insulation performance of the insulator sheds at the grounding end is stronger than that in the middle part.

According to the above results, the wet flashover voltage and pollution flashover voltage of the samples were much smaller than the value of the flashover voltage of the new sample, which indicates that the sample cannot guarantee fine electrical performance in wet environments and salt-fog environments. It is consistent with the above hardness and hydrophobicity test results. Comparing the samples along the string, the aging degree of the high-voltage end of a composite insulator is the most severe, followed by the middle part, and then the grounding end.

### 3.3. Microscopic Characteristics

#### 3.3.1. TGA Analysis

**Figure 4:**
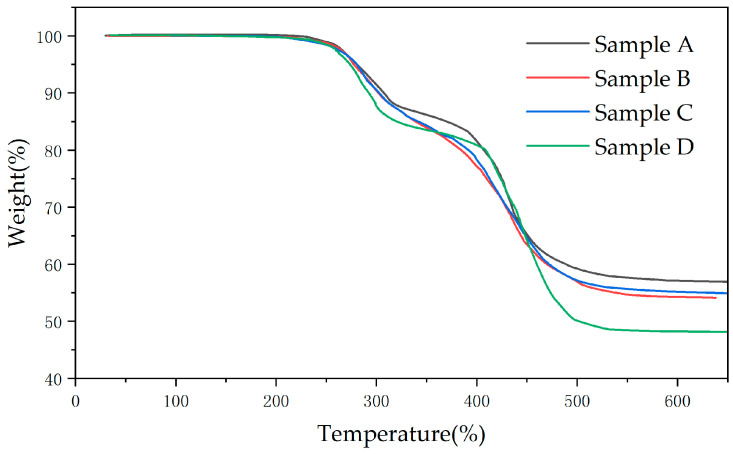
Thermogravimetric curves of different samples.

There are three main components in composite insulator sheds: PDMS, flame retardant Al(OH)_3_, and the reinforcing agent silica. As shown in Figure 4, the decomposition temperature of Al(OH)_3_ is the lowest, and it begins to dehydrate to produce alumina at about 230 °C, which is the first stage of weight loss. When the temperature is higher than 350 °C, the degradation of PDMS is the main degradation in SIR, which is the second stage of weight loss. Silica and other inorganic fillers are still difficult to degrade at high temperatures of 600 °C. Therefore, when the temperature exceeds 640 °C, only inorganic substances that are difficult to decompose are left in the sample, and the quality is almost no longer changed.

Table 7 shows the weight loss and remaining mass of the two stages. According to Table 7, the weight loss in both the first stage and the second stage of pyrolysis of the aging sample is lower than in sample D. It indicates that the charged insulator shed operating for a long time undergoes ATH decomposition and PDMS cracking. This is consistent with the results of the FTIR analysis. The final remaining mass of pyrolysis increases with the above changes. The final remaining mass of pyrolysis can be used to evaluate the aging status of silicone rubber. It can be seen that sample A has the highest remaining mass, an increase of 9.66% compared to the new sample, and the aging is the most severe. The reason is that the high electric field causes ATH to dissolve or decompose at high temperatures, while the PDMS chain of silicone rubber is severely disrupted.

#### 3.3.2. FTIR Analysis

According to different wave numbers, the corresponding functional groups were found, and the peak values of the samples were compared. The corresponding wave numbers of various functional groups are shown in Table 8.

**Figure 5:**
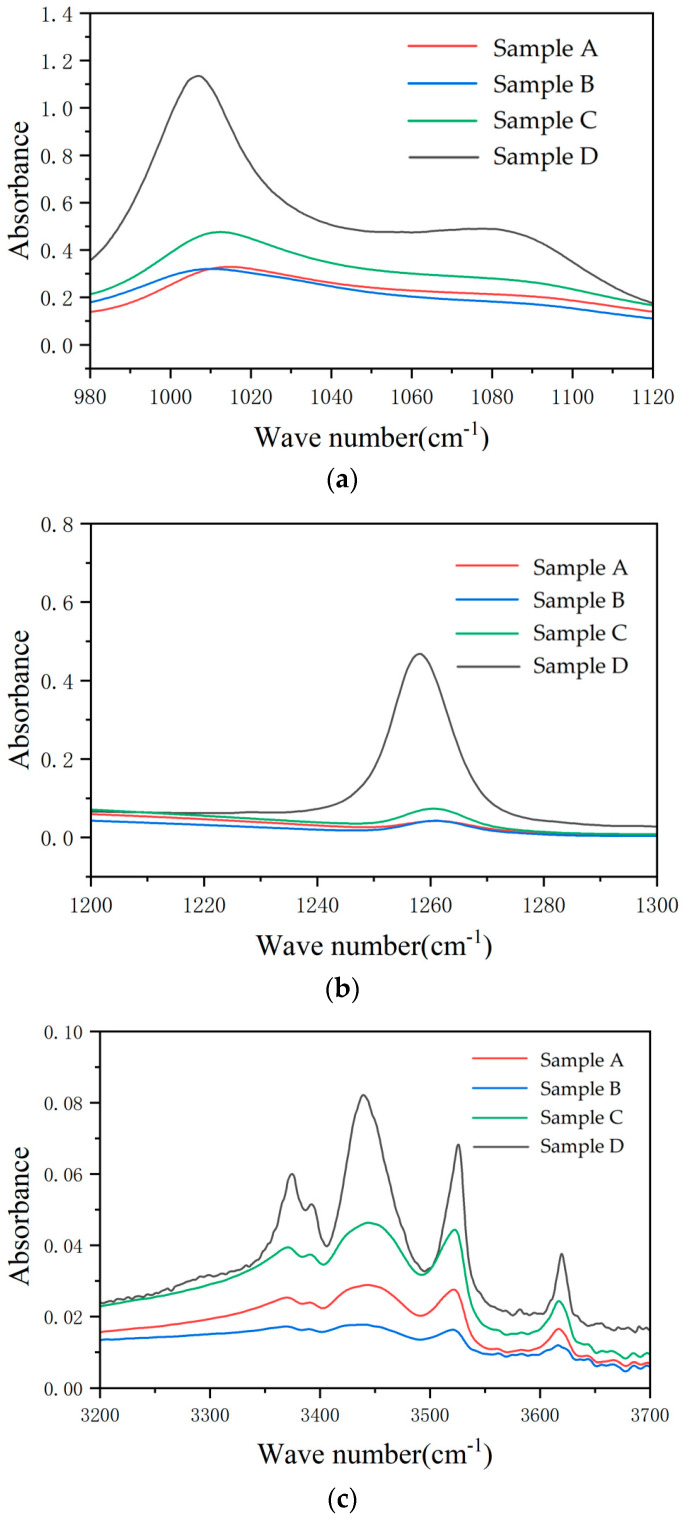
Infrared spectra of different samples: (**a**) the wave number is 1000–1200 cm^−1^, corresponding to the functional group of the main chain Si-O-Si; (**b**) the wave number is 1255–1270 cm^−1^, corresponding to the functional group of the C-H of side-chain Si-CH_3_; (**c**) the wave number is 3200–3700 cm^−1^, corresponding to the functional group of the -OH in filler ATH.

FTIR analysis and comparison of samples A, B, C, and D were carried out, as shown in Figure 5.

According to the above results, it can be seen that the sample is consistent with the band position corresponding to each peak of the new SIR sheet, indicating that no new absorption peak is generated. Aging significantly decreases the absorbance of each peak of the insulator sheet.

The reduction in -CH_3_ indicated that the macromolecular chain is broken, and the reduction in the non-polar chemical bond C-H also further weakens the hydrophobicity. The degree of -CH_3_ damage can reflect the degree of aging to a certain extent. The Si-O-Si chemical bond is broken, and the small silicone molecules are lost, which reduces the absorption peak. The inorganic silicon precipitated on the surface of SIR will lead to an increase in the hardness of the shed, which is consistent with the above mechanical property test conclusions.

The reason for the decrease in -OH on the surface may be that the ATH filler on the surface of SIR is seriously lost due to the loss of the skeleton that can be attached due to the rupture of the PDMS main chain, or it is consumed during long-term operation and dehydrated; however, on the other hand, after the main bond is broken, a certain amount of Si-OH will be formed, reducing the transmittance of the characteristic peak in the wave number range of 3200 to 3700 cm^−1^. According to the comprehensive analysis, the consumption of flame-retardant ATH is the main reason for the decrease in the relative content of -OH.

## 4. Conclusions

In this paper, key parameters such as mechanical properties, electrical properties, and the microstructure of the shed along the string of composite insulators in 500 kV (DC) operation were studied. The experimental results were compared with a new insulator sample and the current standard. The main conclusions can be drawn as follows.

The roughness of the area with thick natural pollution is small, whereas the chalking areas showed significantly higher roughness, where the maximum *Ra* value is 27.131. FTIR analysis reveals that aging causes the main chain of PDMS to break, damaging its skeleton structure and causing filler loss, which is the reason for the increase in hardness of the insulator shed. In addition, the tensile mechanical properties of the test insulator shed failed to meet the operating requirements. Moreover, the hydrophobicity is significantly reduced because the hydrolysis reaction increases the hydrophilic group-OH and decreases the non-polar group -CH3, which leads to polarized insulator sheds.

Under the dry flashover state, the flashover characteristics of the aged insulator do not decrease much, while the samples could not show fine electrical properties in wet environments and salt-spray environments, which is consistent with the results of the hardness and hydrophobicity tests. The flashover voltage of wet flashover and pollution flashover of the grounding end is higher than that of the middle part, which is speculated to be related to the strong insulation performance of the insulator shed itself and the loss of filth and pulverization layer in the wet environment. The breakdown strength of the sample at the high-voltage end is the lowest, which is only 53% of the new sample. The high-voltage end sample can no longer meet the standard requirements, while the breakdown strength of the middle and grounding-end samples could still meet the standard’s requirements.

Natural pollution and chalking exist on the surface of the insulator sheds. The aging degree of the high-voltage end is the most serious. Aging leads to a decrease in the properties of SIR, including increased hardness, reduced hydrophobicity, reduced mechanical properties, and decreased electrical properties. It has an important reference value for insulator life prediction and operation protection. As for suspended insulators, their aging characteristics may be different from those of dead-end insulators, so these differences need to be considered when formulating maintenance strategies.

## Figures and Tables

**Table 1:** Surface roughness testing results. The first number in square brackets means the minimum roughness average (*Ra*) value of the tested distinct areas, and the second number is the maximum value of *Ra*.

Sample	*Ra*/μm
A	[2.435, 20.723]
B	[4.299, 24.011]
C	[3.028, 27.131]
D	[0.712, 0.841]

**Table 2:** Insulator hardness test results. The first number in square brackets means the minimum hardness value of the tested areas, and the second number is the maximum value of hardness.

Sample	A	B	C	D	DL/T 864-2004 [28]
Hardness/HA	[83.75, 85.25]	[81.25, 85.00]	[78.92, 84.33]	[65.2, 67.3]	≥50

**Table 3:** Summary of physical characteristics of insulators. The first number in square brackets means the minimum tensile strength and elongation value of the tested distinct areas, and the second number is the maximum value.

Sample	Tensile Strength/(MPa)	Tensile Elongation (%)
A	[2.41, 2.49]	[116.2, 117.0]
B	[2.47, 2.59]	[133.0, 135.8]
C	[2.71, 2.75]	[144.3, 146.7]
DL/T 810-2012 [24]	≥4	≥150

**Table 4:** Natural pollution test results.

Sample		*ESDD* (mg/cm^2^)	*T/B*	*NSDD* (mg/cm^2^)	*T/B*
A	Top	0.0732	1.32	3.0842	1.11
	Bottom	0.0553		2.7719	
	Average	0.0642		2.9280	
B	Top	0.0449	1.05	1.0448	0.70
	Bottom	0.0427		1.4956	
	Average	0.0438		1.2702	
C	Top	0.0580	1.33	1.9477	1.08
	Bottom	0.0437		1.7975	
	Average	0.0508		1.8726	

**Table 5:** Insulator breakdown test result.

Sample	A	B	C	D	GB/T 1695-2005 [29]
*E_b_* (kV/mm)	11.248	14.112	16.335	21.316	≥14
σ (%)	6.5	7.1	5.6	3.7	-

**Table 6:** Flashover test results.

Sample	Dry Flashover		Wet Flashover		Pollution Flashover		Breakdown	
	*E_f_* (kV/mm)	*σ* (%)	*E_f_* (kV/mm)	σ/%	*E_f_* (kV/mm)	σ/%	*E_f_* (kV/mm)	σ/%
A	0.546	1.5	0.185	2.7	0.107	3.4	11.248	6.5
B	0.554	0.9	0.204	3.0	0.110	5.8	14.112	7.1
C	0.547	1.7	0.230	5.4	0.124	9.1	16.335	5.6
D	0.559	0.6	0.372	2.1	0.224	2.5	21.316	3.7

**Table 7:** Weight loss and remaining mass in two thermal weight-loss stages.

Sample	First-Stage Weight Loss (%)	Second-Stage Weight Loss (%)	Residual Mass (%)
A	12.64	29.68	57.68
B	15.48	31.16	53.36
C	15.48	29.40	55.12
D	17.70	34.28	48.02

**Table 8:** FTIR absorption bands of HTV SIR materials.

Wave Number (cm^−1^)	Functional Group
1000–1100	Main chain Si-O-Si
1255–1270	C-H of side-chain Si-CH_3_
3200–3700	-OH in filler ATH

## Data Availability

The original contributions presented in this study are included in the article, further inquiries can be directed to the corresponding author.
